# Items, News and Gossip

**Published:** 1872-03

**Authors:** 


### Items, News and Gossip.

M. Maisonneuve, of the Hotel Dieu, makes large use of ice-poultices in the treatment of wounds. Lumps of ice the size of large marbles are scattered at intervals of an inch or more over a tolerably thick linseed-meal poultice, the whole is then covered with a little more of the meal and a cloth, and the thick side is applied to the wound.—— The use of extract of conium in the treatment of inflammation of the breast is being revived in Germany.—— Prof. Deneffe, of Ghent, has used for a couple of years, a combination of camphor and bromine which he thinks a most excellent sedative for the nervous system. "Monobromized camphor" differs from ordinary camphor by the substitution of one atom of bromine for one of hydrogen.—— In *pruritus vulvæ*, Mr. McGrath (*Canada Lancet*) states that he has found this lotion, applied by means of a soft sponge after ablution, morning and evening, very satisfactory:

| | | |
|---|---|---|
| R. | Biborate of Soda, | dr. ij; |
| | Hydrochlorate of Morphia, | gr. xx; |
| | Hydrocyanic Acid, | dr. j; |
| | Glycerine, | oz. j; |
| | Distilled rose water, | oz. viij. |
| M. | | |

—— Dr. Green (*Atlanta Med. and Surg. Jour.*) lauds permanganate of potash as a local application in carbuncle. It is nearly painless, and after a few moments the application produces comfort. After the slough is discharged he dresses the part with carbolic acid lotion, and spreads over all, lint smeared with benzoated zinc ointment.—— Dr. Schmidt (*N. Y. Med. Jour.*) recommends one drop of tincture of iodine every two hours in the incontinence of urine of advanced life.—— Dr. Monteverdi, a Venetian physician, asserts that Peruvian bark and its preparations possess emmenagogue properties superior to ergot. Sulphate of quinia, five grains every hour, will bring on miscarriage and premature birth. In protracted labor with feeble pains, he uses this instead of ergot, and finds it rapidly successful. So also in rigidity of the os and retention of the placenta.—— Dr. Walker (*Med. Archives*) reports a case of incipient bone felon cured by congelation. He placed the finger in a freezing mixture, equal parts of snow and salt in a tumbler until sensibility ceased, repeating the process as it returned, continuing for two hours. Perfect cure resulted.—— Dr. Bird (*Med. and Surg. Reporter*) uses an ointment of charcoal and lard to prevent pitting in small-pox. —— The *Georgia Med. Companion* recommends a solution of extract belladonna and glycerine (dr. j to oz. j) for local application, instead of the ordinary ointment or plaster.—— Prof. Gross characterizes carbolic acid as a fanciful preparation that has had its day. But the Edinburgh Med. Journal adds another to its long list of uses, in that in gunpowder burns, in addition to its antiseptic and anæsthetic properties, it has the power of suspending the carbon in solution and withdrawing it from the skin, thus preventing discoloration.—— Dr. Caspari has for twenty-five years treated obstinate vomiting successfully with tincture of iodine. —— Dr. Pattee, of Boston, gives in the vomiting of pregnancy, from dr. j to oz. ss. of the acid syrup of the hypophosphite of lime.—— In 1820 there were only 100 dentists in the United States. In 1858 there were 4,000. —— Thirty-one physicians have been made baronets in England since 1645.—— The cost of the clergy to the United States is estimated at $12,000,000 annually ; that of the criminals, $40,000,000, and of spirits, $600,000,000. So says the Medical Record.—— *The Clinic* (Cincinnati) is proving itself a success. We congratulate editors and publishers.—— Dr. Both proposes to stamp out small-pox by salt diet, but the chief physician of Iceland claims to have smoked it out with sulphur, with the aid of sulphurous acid and water drank by the patients. The disease at all events disappeared.—— Dr. Toner claims to have established by statistics, that the average American mother has fallen off as a producer. He says only one-half as many children are born now to each thousand women as in 1800, and that each decade shows a regular decrease.—— Over a million and a quarter of dollars worth of opium were imported through the custom house of New York in 1871.—— The *lobelia inflata*, a drug much praised and abused by quacks, and somewhat slighted by the profession, is in constant use among the out-patients of the city hospital, for diseases of the chest. In doses of ten minims, three times a day, it appears frequently to produce the most admirable effects in cases of chronic bronchitis, complicated with a tendency to paroxysmal asthma. It is commonly given in conjunction with sedatives, expectorants or stomachics, often agreeing remarkably well with the latter. Patients taking it frequently complain of nausea and sense of depression during the half-hour or so following each dose, but it seems on the whole to decidedly improve the appetite and digestion. If the nausea be excessive, combination with a few drops of dilute hydrocyanic acid is often used.—*Cincinnati Lancet.* —— Delay in appearance of the JOURNAL enables us to announce the death upon the same day (April 5th) of Zina Pitcher, M.D., of Detroit, Mich., and Samuel Jackson, M.D., of Philadelphia. Dr. Pitcher was aged about 75 years, was one of the oldest citizens, assisted in founding the free schools of Detroit, and the State University of which he was for many years a regent. He had occupied many prominent secular and professional positions. He was an Emeritus Professor in the Michigan University, a position to which he was nominated by the present writer. He had also been one of the Presidents of the American Med. Association. He was a model of the courtly gentleman of the old school, and his death will be widely lamented. Dr. Jackson was in his 85th year, and had been connected with the University of Pennsylvania for 28 years, the latter portion of the time as an Emeritus Professor. Few professional men in our country were more extensively known or more highly esteemed. —— Dr. Toner, of Washington, the most indefatigable of statisticians, is compiling a Medical Directory and Register of the United States. Any medical gentleman wishing immortality should communicate his name and *status* at once, or forever after hold his peace. Dr. S. W. Butler, of the *Philadelphia Reporter*, will receive names and addresses. The Directory will cost $5.00 till May, and afterwards $6.00.

